# Cloning and characterization of a pyrethroid pesticide decomposing esterase gene, *Est3385*, from *Rhodopseudomonas palustris* PSB-S

**DOI:** 10.1038/s41598-018-25734-9

**Published:** 2018-05-09

**Authors:** Xiangwen Luo, Deyong Zhang, Xuguo Zhou, Jiao Du, Songbai Zhang, Yong Liu

**Affiliations:** 1 Key Laboratory of Pest Management of Horticultural Crop of Hunan Province, Hunan Plant Protection Institute, Hunan Academy of Agricultural Science, Changsha, 410125 China; 2 Department of Entomology, University of Kentucky, Lexington, KY 40546 USA

## Abstract

Full length open reading frame of pyrethroid detoxification gene, *Est3385*, contains 963 nucleotides. This gene was identified and cloned based on the genome sequence of *Rhodopseudomonas palustris* PSB-S available at the GneBank. The predicted amino acid sequence of Est3385 shared moderate identities (30–46%) with the known homologous esterases. Phylogenetic analysis revealed that Est3385 was a member in the esterase family I. Recombinant Est3385 was heterologous expressed in *E. coli*, purified and characterized for its substrate specificity, kinetics and stability under various conditions. The optimal temperature and pH for Est3385 were 35 °C and 6.0, respectively. This enzyme could detoxify various pyrethroid pesticides and degrade the optimal substrate fenpropathrin with a *K*m and *V*max value of 0.734 ± 0.013 mmol·l^−1^ and 0.918 ± 0.025 U·µg^−1^, respectively. No cofactor was found to affect Est3385 activity but substantial reduction of enzymatic activity was observed when metal ions were applied. Taken together, a new pyrethroid degradation esterase was identified and characterized. Modification of Est3385 with protein engineering toolsets should enhance its potential for field application to reduce the pesticide residue from agroecosystems.

## Introduction

Synthetic pyrethroids have been used extensively worldwide to control insect pests damaging cereal, potato, cotton, and fruit crops^1^. To date, synthetic pyrethroids have been used in field for more than 30 years due mainly to their high toxicities to insect pests, allowing each field application with a relatively low dosage^2^. Furthermore, these pesticides were reported to be less toxic to mammals and thus became the top choices of agricultural industries to replace other more toxic pesticides, including organochlorine and organophosphorus pesticides.

However, the ever increasing use of synthetic pyrethroids in the agro-ecosystem has raised significant concerns in the recent years. Synthetic pyrethroids could be highly toxic to non-targeted organisms such as ladybeetles (*Cycloneda sanguinea* L. and *Harmonia axyridis* Pallas)^3^, bees (*Apis mellifera*)^4^, and arthropods and vertebrates in the aquatic subsystem^5^. Moreover, some synthetic pyrethroids were reported to have neurotoxicity^6^, reproductive toxicity^7^, cytotoxicity^8^, or teratogenicity and carcinogenicity^9^. These public concerns promoted us to investigate the potential routes that can be used to reduce the amount of synthetic pyrethroid residues in the agro-ecosystem to provide a more safe and healthier environment for agricultural industry.

Residues of synthetic pyrethroids can be broken down by both biotic and abiotic approaches. For biotic approaches, synthetic pyrethroid residues can be decomposed through degradation by microbes. To date, multiple soil resident bacteria have been identified to have the abilities to degrade synthetic pyrethroid residues and several bacterial genes controlling this function are cloned and characterized^10^. These enzymes would be play a potential application in subsequent detoxification of the synthetic pyrithriod residues.

Several photosynthetic bacteria in genus *Rhodopseudomonas* have been demonstrated to have the ability to degrade various synthetic pyrethroids^11^. In 2009, Zhang and his colleagues cloned a gene encoding 2OG-Fe(II) oxygenase from *Rhodopseudomonas* sp. PSB07-21 and showed that this enzyme was capable of degrading synthetic pyrethroids^12^. So far, no esterase gene from genus *Rhodopseudomonas* has been reported to have the ability to degrade synthetic pyrethroids.

In this study, we identified and cloned an esterase gene from *R. palustris* PSB-S after searching the existing genome sequences of this strain deposited at the NCBI database. This identified gene was cloned and expressed in *E. coli* Rosetta cells. Analysis of this recombinant esterase (i.e., Est3385) indicated that this enzyme was indeed capable of decomposing various synthetic pyrethroids, and fenpropathrin as its optimal substrate. We consider that this enzyme can play a significant role in detoxification of pyrethroid residues in field.

## Methods

### Sources of bacteria strains, and cloning and expression vectors

*Rhodopseudomonas palustris* strain PSB-S was isolated, characterized and stored in our lab. *Escherichiacoli* strain Rosetta and the cloning vector pMD-18T were purchased from Takara Biotechnology (Dalian, China). The expression vector, pGEX-3x, was from Dr. Jian Yan (Virology and Biotechnology Institute, Zhejiang Academy of Agricultural Sciences, Hangzhou, China). For expression of the recombinant protein, *E. coli* Rosetta was cultured in Luria-Bertani medium containing proper antibiotics. Culture medium for *R. palustris* PSB-S contained 0.2 g K_2_HPO_4_, 0.8 g KH_2_PO_4_, 0.2 g MgSO_4_, 0.1 g CaSO_4_·2H_2_O, 0.0033 g NaMoO_4_·2H_2_O, 0.005 g FeSO_4_·7H_2_O, and 1.5 g yeast extract in one liter H_2_O, pH 7.2.

### Chemical reagents and restriction enzymes

Technical-grade synthetic pyrethroids were all purchased from the Hainan Zhengye Pesticide Chemical Co., Ltd (Hainan, China). Other grades of synthetic pyrethroids were from the Tianjin Orient Green Technology and Development Co., Ltd (Tianjin, China). All endonucleases and reagents used in the study were from the Transgen Biotech (Beijing, China). Chemicals used for the analytical analyses were analytical grades and were purchased from the Shanghai Sango Biological Engineering Technology & Services Co., Ltd (Shanghai, China).

### Cloning, expression, and analysis of recombinant *Est3385* gene

Sequences of three published synthetic pyrethroids degrading esterases (e.g., pyrethroid-hydrolyzing carboxylesterase pytH)^13^, pyrethroid-hydrolyzing enzyme Sys410 (sys410)^14^, and esterase (pytZ)^15^ were aligned and the conserved sequence regions were used to blast search for new candidate synthetic pyrethroids degrading esterase genes in the genome of *R. palustris* strain PSB-S deposited at the GenBank (www.ncbi.nlm.nih.gov). Nucleotide sequences and amino acid sequences obtained initially were aligned using the MEGA 6.0 software^16^ and then compared with other known esterase and lipase sequences found in different pyrethroid degrading bacterial strains to identify the sequence of esterase *Est3385* in *R. palustris* strain PSB-S. The deduced molecular weight of Est3385 was estimated using the ExPASy software (http://web.expasy.org/compute_pi/). The protein structure of EST3385 was predicted and constructed with homology-modelling (https://www.swissmodel.expasy.org/).

The resulting full length open reading frame of *Est3385* was PCR amplified using primers est3385-F and est3385-R (est3385-F 5′-CGCGGATCCGCATGAAGGACGTGGCG-3′ and est3385-R 5′-CCGGAATTCCGssCATCGGCCGGTAGGT-3′, the underlined sequences are the *Bam*H I and *Eco*R I restriction sites). The amplified PCR fragment was digested with *Bam*H I and *Eco*R I restriction enzymes, and inserted into the pre-digested *Bam*H I/*Eco*R I site in the pGEX-3x vector to produce plasmid pGEX-3x-3385. The plasmid DNA was transformed into *E. coli* Rosetta cells, and the recombinant protein was produced and then purified using the Glutathione Sepharose 4B Kit as instructed (GE Healthcare, Beijing, China). Quality of the purified Est3385 recombinant protein was analyzed in SDS-PAGE gel. Concentration of the purified recombinant protein was estimated by the BCA method using bovine albumin as a standard control^17^. To confirm the recombinant protein, protein bands in the SDS-PAGE gels were transferred to PVDF membrane followed probing using an anti-GST antibody diluted 1:1000 (v/v) in a PBS buffer. The detection signal was visualized using nitro blue tetrazolium and 5-bromo-4-chloro-3-indolyphosphate (BCIP).

### Substrate specificity and kinetics assays

Synthetic pyrethroids, ρ-nitrophenyl esters and short-chain fatty acids were used as substrates for substrate specificity assays. Est3385 was tested for its hydrolytic capacity on different synthetic pyrethroids, ρ-nitrophenyl esters and short-chain fatty acids according to a previously published method^18,19^. Treatments with glutathione S-transferase (GST) were used as a control.

Enzyme kinetics of Est3385 was determined using five different concentrations (10 µg · ml^−1^ to 50 µg · ml^−1^) of fenpropathrin as the substrate, and the kinetic constant of Est3385 was calculated using the Michaelis-Menten equation published previously^20^. For each treatment, the final non-degraded fenpropathrin was less than 10% of the total fenpropathrin added to the reactions. Each experiment was repeated three times.

### Effects of temperature and pH value on enzyme activity

To investigate the effect of temperature on Est3385 activity, we incubated purified recombinant Est3385 protein with fenpropathrin in 0.1 µmol·ml^−1^ PBS buffer, pH 7.2, at different temperatures (e.g. 15, 25, 35, 45, 55 and 65 °C). For thermos stability assays, the mixed Est3385 and fenpropathrin samples were incubated first at 35 °C for 1 h followed by 5 min in a 100 °C water bath to end the reaction. The treated samples were then tested again for the residual, and the results were compared with the activity of non-pretreated Est3385. The residual activity of the enzyme was presented as the percentage of the activity from the pretreated verses the non-pretreated Est3385 enzyme. The effect of different pH value on the Est3385 activity was determined by conducting the activity assays in PBS buffers adjusted to various pH values (e.g., pH 4.0, 5.0, 6.0, 7.0, 8.0 and 9.0) for 1 h. For all the assays, reactions in the absence of Est3385 enzyme were used as controls. Each experiment was repeated three times.

### Effect of metal ions and chemical regents on Est3385 activity

Metal ions were added to a PBS buffer, pH 7.2, to produce PBS solutions containing Na^+^, K^+^, Mg^2+^, Zn^2+^, Cu^2+^, Mn^2+^ or Fe^3+^ (1 µmol·ml^−1^ each). Several other reagents were also added to the PBS solution to produce PBS solutions with 1.0% Tween-80, 1.0% Tween-20, 1 µmol·ml^−1^ ethylenediaminetetraacetic acid (EDTA), 1 µmol·ml^−1^ β-mercaptoethanol, 1 µmol·ml^−1^ phenylmethane sulfonyl fluoride (PMSF) or 1 µmol·ml^−1^ diethyl pyrocarbonate (DEPC). Purified recombinant Est3385 (1 μg·ml^−1^) was then added to each reaction and the reactions were incubated at 35 °C for 10 min. During these assays, reactions without addition of a metal ion or a chemical reagent were used as controls. Activity of the enzyme in each reaction was calculated as the percentage between the enzyme activity found in a specific reaction versus that from its control. Each experiment was repeated three times.

### Gas chromatography (GC) analysis

The residual pyrethroid pesticides, ρ-nitrophenyl esters and short-chain fatty acids left in individual reactions were quantified by GC as described previously^19,21^. Briefly, samples from above assays were extracted individually three times with n-hexane (10 ml each time). The organic phase from a sample was filtrated using the SampliQ Florisil PR 1GM 6 ML as instructed (Agilent Technologies, CA). The resulting residue was dissolved in n-hexane and then quantified by an Agilent 6890N system equipped with a HP-5 chromatographic column (30 m × 0.32 mm × 0.25 µm) as instructed (Agilent, USA). Temperatures set for the injection port and the electron capture detector (µECD) were at 250 °C and 320 °C, respectively. The pipe was kept at 160 °C for 5 min and then ramped at 10 °C·min^−1^ to 200 °C for 1 min. Subsequently, the pipe was ramped again at 10 °C·min^−1^ to 280 °C for another 8 min. High purity nitrogen gas (>99.999%) was introduced at 1 ml·min^−1^ and the injection volume was 1 µl. For the concentrations of pesticide substituents, ρ-nitrophenyl esters and short chain fatty acids, the peak areas mantra was used to analyze gas chromatography using the known corresponding standards as controls.

## Results

### Identification of Est3385 through Sequence Analysis

**Figure 1:**
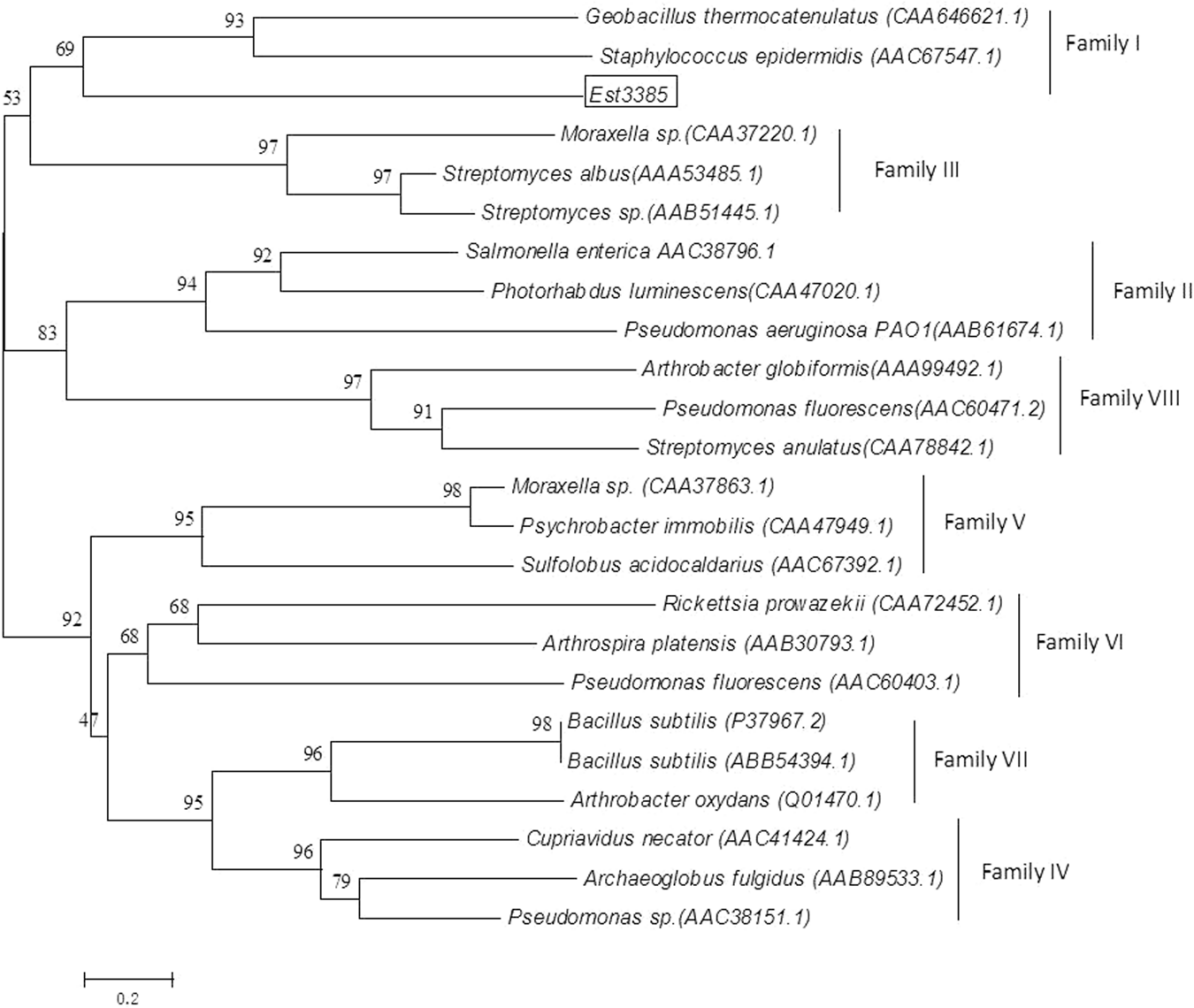
Phylogenetic relationship between Est3385 and other proteins belonging to different esterase families. The number in the brackets are the accession numbers of individual protein sequences, while the boxed sequence is Est3385.

Conserved sequences from three known pyrethroid pesticide degrading esterase were identified through sequence alignment, and were used to search new candidate esterase genes in a pyrethroid pesticide decomposing bacteria, *R. palustris* strain PSB-S. A total of six candidate genes (gene ID: PSB-SGL000612, PSB-SGL000613, PSB-SGL000878, PSB-SGL003706, PSB-SGL003752, and PSB-SGL0003385) were found and gene PSB-SGL0003385 (referred to *Est3385* thereafter) was selected for further analysis. Result of phylogenetic analysis of *Est3385* is shown in Fig. 1. The full length open reading frame of *Est3385* contains 963 nucleotides and is now deposited in the GenBank (accession number: KU377526). Although the conserved region in *Est3385* sheared 53.2–76.2% nucleotide sequence similarities with the sequences of the three known pyrethroid pesticide degrading esterase genes [e.g., *pytH*, *pytZ* and *est843-sys410*, Fig. S1), the protein sequence of Est3385 sheared only 24.1% sequence identity with pytH, 13.9% identity with pytZ, and 20% identity with est843-sys410 (Fig. S2). Phylogenetic analysis indicated that *Est3385* belonged to the esterase family cluster I, and was together with an esterase from *Geobacillus thermocatenulatus* (CAA64621.1) and an esterase from *Staphylococcus epidermidis* (AAC67547.1). The 3D structure of protein Est3385 contains six α-helixes and nine β-sheets (Fig. S6).

### Expression and purification of the recombinant Est3385

**Figure 2:**
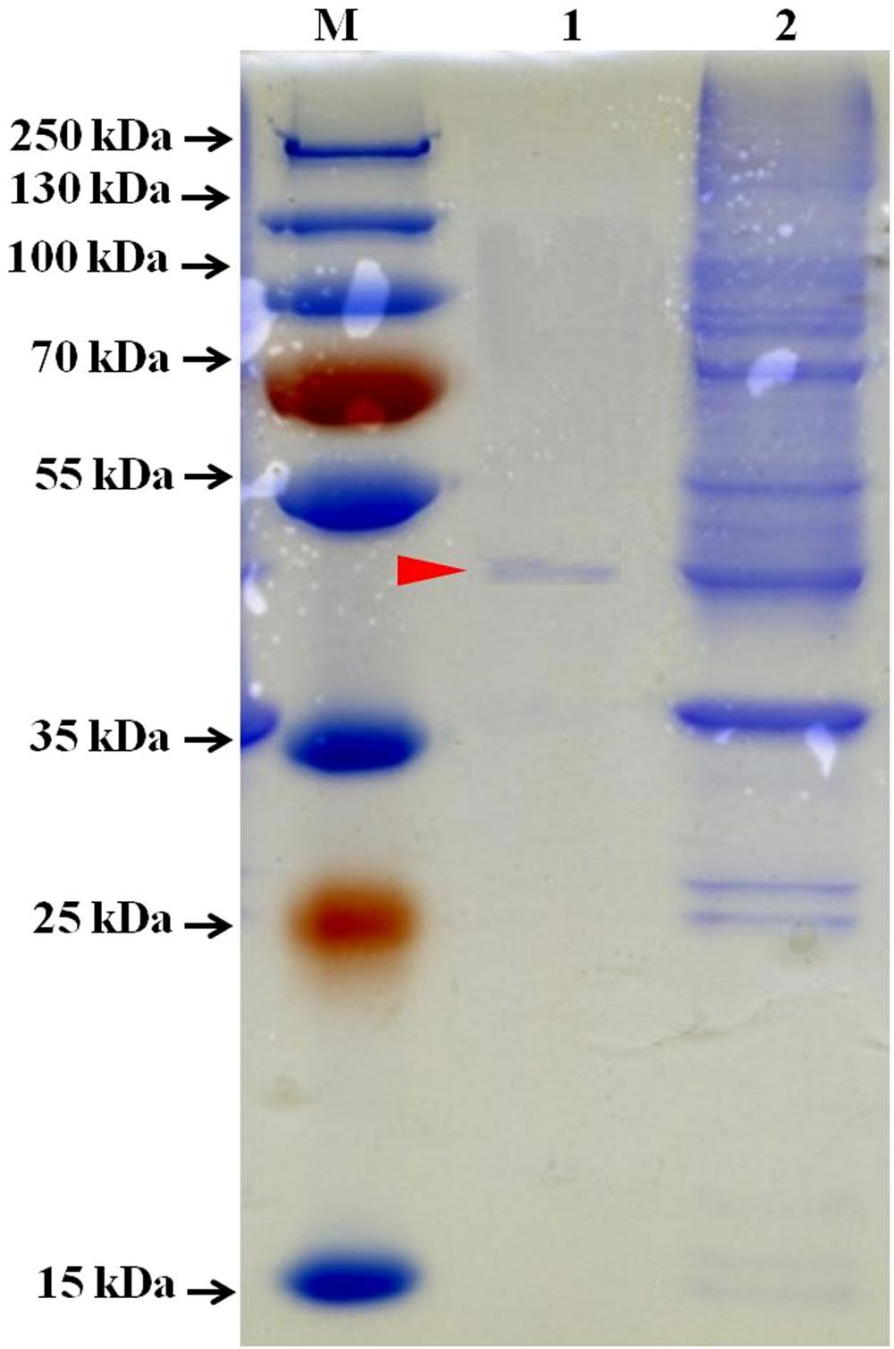
Expression, purification and SDS-PAGE of the recombinant protein Est3385. M: protein marker; lane 1: purified Est3385 protein; lane 2: total protein from *E. coli* Rosetta harboring pGEX-3x-3385.

Coding sequence of *Est3385* was cloned into the pGEX-3x expression vector and the recombinant Est3385 protein was expressed in *E. coli* Rosetta. The expressed recombinant protein was then purified by affinity chromatography and examined in SDS-PAGE gels (Fig. 2). A single protein band of approximately 59 kDa was found in lanes loaded with the soluble protein fraction or purified recombinant Est3385 protein. Expression of the recombinant protein was then confirmed by probing the membrane with an anti-GST antibody. Based on the size of protein markers, the molecular mass of this recombinant protein was similar to the estimated molecular mass for Est3385 (33.94 kDa plus ~26 kDa GST tag, Fig. S3).

### Substrate specificity and enzyme kinetics of Est3385

**Figure 3:**
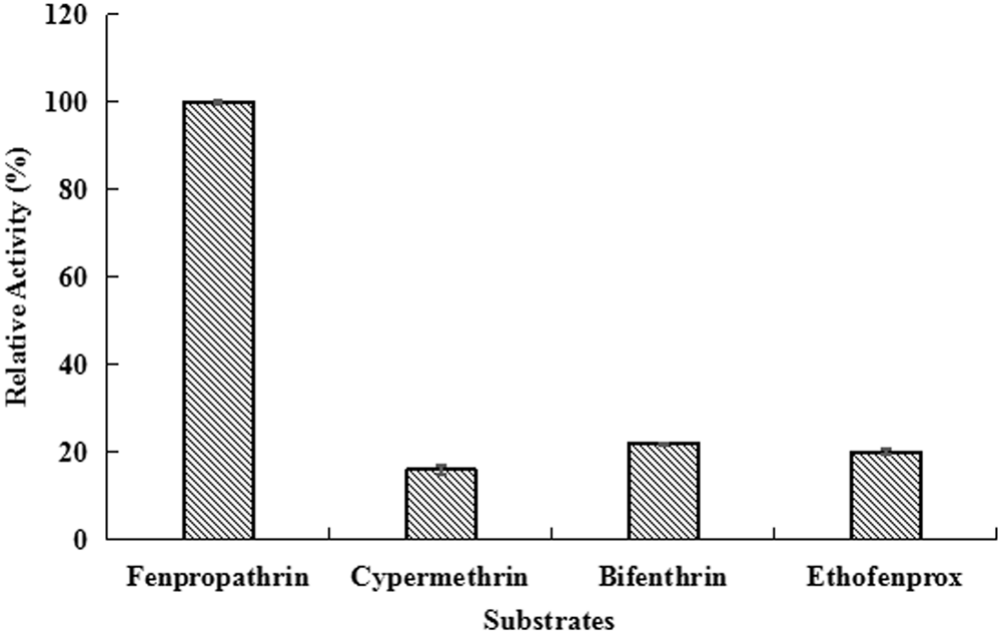
Substrate specificity analysis for purified Est3385 protein. The substrate activity was set as 100% for reactions with fenpropathrin as the substrate. Each result is the mean of three independent experiments ± standard deviation.

Substrate specificity was determined using various pyrethroid pesticides, ρ-nitrophenyl esters, and short-chain fatty acids in the assays. The results shown in Fig. 3 indicated that although Est3385 could degrade all the four tested pyrethriod substrates, fenpropathrin was the most sensitive substrate for Est3385. In this study, control treatments using only GST protein or no enzyme was added did not yield a clear degradation of any of the assayed substrates (Fig. S4). In this study we also determined that Est3385 was able to degrade ρ-nitrophenyl acetate and formic acid effectively. This enzyme had, however, very little effect on ρ-nitrophenyl butyrate and ρ-nitrophenyl caproate, and isobutyric acid (Fig. S5). Our enzyme kinetic studies using fenpropathrin as a substrate showed that the kinetic constants of *K*m and *V*max were at 0.734 ± 0.013 mmol·l^−1^ and 0.918 ± 0.025 U·µg^−1^, respectively (Fig. S7).

### Effect of temperature and pH value on Est3385 activity

**Figure 4:**
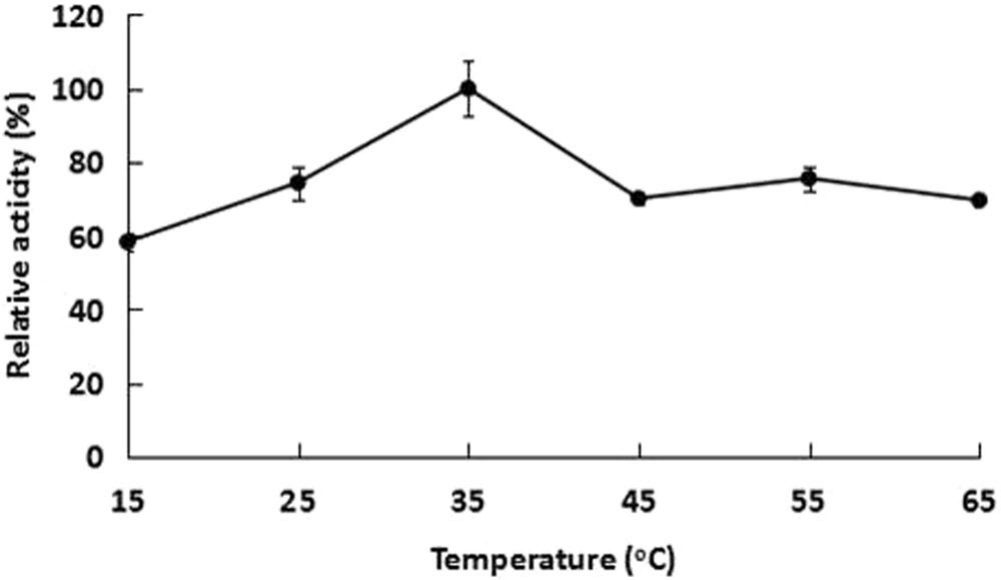
Temperature effect on the activity of purified Est3385 protein. The highest enzyme activity was observed at 35 °C and was set as 100%. Each result is the mean of three independent experiments ± standard deviation.

**Figure 5:**
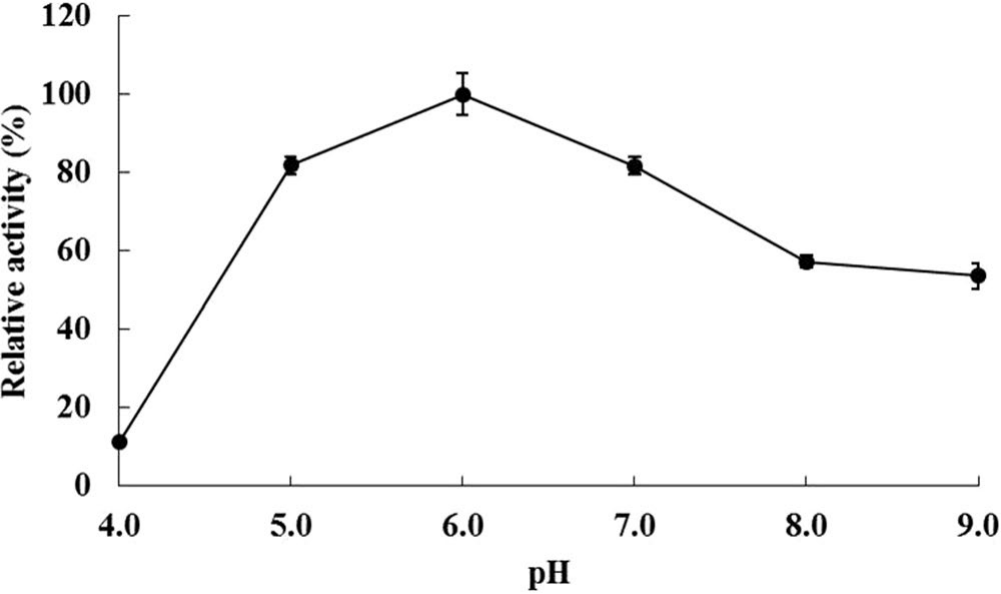
Effect of pH value on the activity of purified Est3385 protein. The highest enzyme activity was observed at pH 6.0 and was set as 100%. Each result is the mean of three independent experiments ± standard deviation.

When enzyme activity was tested under various temperature conditions, the highest degradation efficiency of Est3385 was observed at 35 °C (Fig. 4). When the reaction temperature was changed to 25, 45, 55 or 65 °C, the degradation efficiency was found to be moderately reduced. Even when the reaction temperature was set at 15 °C, the relative enzyme activity was still at about 60% compared with that observed at 35 °C. Results shown in Fig. 5 indicated that the optimal pH value for Est3385 activity was at 6.0. The results also showed that under the elevated pH conditions (pH 8.0 to 9.0), Est3385 still showed about 50% enzyme activity compared that observed under the optimal pH condition. These observations indicated that Est3385 had a good tolerance to temperature changes and alkaline conditions.

### Effects of metal ions and chemical reagents on Est3385 activity

**Figure 6:**
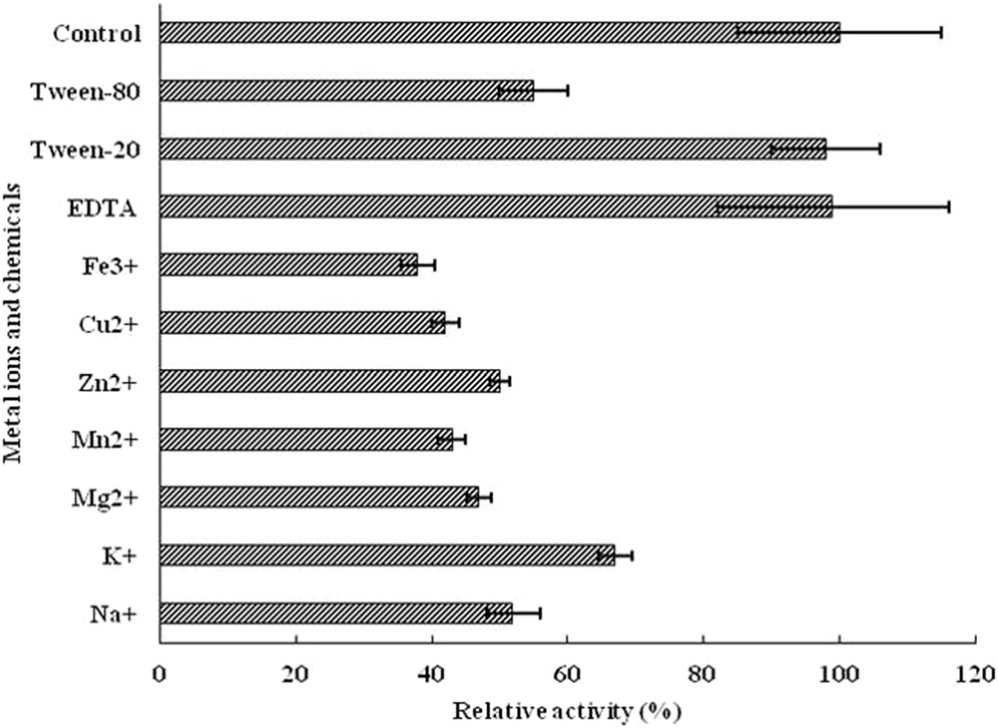
Effect of metal ions and chemical reagents on the activity of purified Est3385 enzyme. Each metal ion was used at a concentration of 1.0 µmol·ml^−1^, and the concentration of each chemical reagent was used at 1.0%. Relative enzyme activity in a reaction without the addition of metal ions and chemical reagents was set at100%. Each result is the mean of three independent experiments ± standard deviation.

To determine if metal ions could affect Est3385 activity, we incubated purified Est3385 with fenpropathrin in a PBS buffer containing various metal ions. We then assayed Est3385 activity in these reactions as described above. Results shown in Fig. 6 indicated clearly that all the metal ions tested could significantly suppress but not completely eliminate the activity of Est3385. Among these six metal ions, Fe^3+^ showed the strongest inhibition effect (~63%) followed by Cu^2+^, Mn^2+^ and Mg^2+^ (~50%). These results demonstrated that the higher the metal ion valence had stronger effect on Est3385 activity. In this study we also tested five chemical reagents for their effects on the Est3385 activity. Results showed that tween-80 could indeed reduce the activity of Est3385 while Tween-20 and EDTA showed no clear effect on the activity of Est3385 (Fig. 6). The assays using protease inhibitor (PMSF) or protease modifier (DEPC or β-mercaptoethanol, Table 1) showed that Est3385 activity was only slightly affected by DEPC, a histidine modifier, but was not affected by PMSF, a serine protease inhibitor, or β-mercaptoethanol, a cysteine modifier (Table 1).

**Table 1:** Effects of amino acid, protease inhibitor or protease modifier on the activity of purified Est3385 enzyme.

Inhibitors/modifiers	Concentration (µmol·ml^−1^)	Relative enzyme activity (±SD)*
Mercaptoethanol	1.0	100 ± 5.36
PMSF	1.0	103.97 ± 7.64
DEPC	1.0	91.83 ± 3.25

*Relative enzyme activity in a reaction without the addition of amino acid, protease inhibitor or protease modifier was set at100%. SD represents standard deviation.

## Discussion

It was reported that the key step involved in degradation of pyrethroid pesticides is the cleavage of the ester-bond in the fenpropathrin compound^13,14,21^. To target this degradation step, pyrethroid hydrolyzing esterase 3 (pye3) was previously identified through direct screening of esterase activities on ester, an indicator commonly used for the esterase activity assays. This esterase screening assay was recommended by the authors as a time-saving approach for identification of pyrethroid hydrolyzing esterases. In addition, this method did not require expensive quantification assays for pyrethroids residues^22^. In the present study, we searched the available genome sequence of *R. palustris* PSB-S using the conserved sequences from three published pyrethroid degradation esterase genes from other bacteria species in the GenBank^13–15^ and then cloned *Est3385* from *R. palustris* PSB-S. Through enzyme kinetics studies, the ability of Est3385to decompose pyrethroid pesticides was confirmed. We consider that this gene cloning and characterization approach should allow us to screen more new candidate genes important for pesticide residue detoxification in a more straight forward and time-saving manner.

Phylogenetic study (Fig. 1) indicated that Est3385 is a member belonging to the esterase family I. Several other characterized pyrethroid pesticide degrading enzymes were also found in the esterase family VI cluster (i.e., pyt Y^18^ and pyt Z^15^) or in the family V cluster (i.e., est843-Sys410^14^). The conserved pentapeptide motif, Gly-X-Ser-X-Gly, was found in many pyrethroid pesticide degrading enzymes^13,14,18^, and residue Ser in this motif was shown as a nucleophile catalyzing the hydrolysis of ester bonds^22^. Because EstP, a pyrethroid pesticide decomposition protein, does not have this conserved motif ^23^, we consider that pyrethroid pesticide degradation enzymes contain other motif(s) may also serve as nucleophile(s). Interestingly, analysis of Est3385 sequence did not identify any conserved pentapeptide motif. Although the amino acid residues Ser117-119 and Ser125-128 exist in surface of protein Est3385 (Fig. S6), the fact that neither PMSF nor DEPC abolished Est3385 activity during the assays (Table 1), indicating that no histidine or serine presented in the active site of Est3385.Thus, the amino acid residue(s) in Est3385 needed for the breakdown of ester bonds in the pyrethroid pesticide compound remained unknown.

Results shown in this paper indicated that Est3385 is a moderate pyrethroid detoxifying enzyme with a deduced molecular mass of 33.94 kDa (Fig. 2). This molecular mass is smaller than that reported for permethrinase (61 kDa) from *Bacillus cereus* SM3^24^, pyrethroid hydrolase (56 kDa) from *Aspergillus niger* ZD11^25^, carboxylesterase (60 kDa) from mouse liver microsome^26^, carboxylesterase E3 (58.6 kDa) from *Nephotettix cincticeps* Uhler^27^, EstP (73 kDa) from *Klebsiella* sp. ZD112^23^, and PytY (41.7 kDa) from *Ochrobactrum anthropi* YZ-1^18^. This molecular mass is, however, bigger than that reported for carboxylesterase (31 kDa) from *Sphingobium* sp. JZ-1^13^, esterase (31.15 kDa) from the metagenome^22^, and carboxylesterase PytZ (24.2 kDa) from *Ochrobactrum anthropi* YZ-1^15^. Similar to other recombinant pyrethroid degrading enzymes, Est3385 was quickly deployed by IPTG at 35 °C. The operational performance contributed to an identification of characteristics and potential utilization for large-scale manufacturing of the recombinant protein.

This enzyme Est3385 showed a remarkable activity and stability at a broad range of temperature (25 to 65 °C) and pH conditions (pH 5 to 8). Our results have indicated that Est3385 could decompose pyrethroids in the absence other cofactors, based on the fact that the activity of Est3385 was not inhibited by chelating reagents (Fig. 6), which similar as the degrading enzyme EstSt7^28^.

Taken together, a new pyrethroid decomposing enzyme has been identified and cloned in this study. The novelty of this newly identified protein is its lack of the conserved pentapeptide motif, Gly-X-Ser-X-Gly, and AT domain^29^, found in other pyrethroids degrading enzymes (Table S1). Utilizing fenpropathrin as asubstrate, we have demonstrated that this novel enzyme is thermostable and can tolerate a wide range pH conditions. We propose that Est3385 is a potential pyrethroid pesticides decomposing enzyme for agricultural industry. It is noteworthy that Est3385 can be moderately inhibited by metal ions (Fig. 6), a potential limiting factor for commercial application of this enzyme in field. It is, however, reasonable to speculate that modification of this enzyme through current biotechnologies should eliminate or at least reduce this barrier to minimal as previously discussed^30^.

## Electronic supplementary material

dataset 1
