# Supplementary material for: Cloning and characterization of a pyrethroid pesticide decomposing esterase gene, Est3385, from Rhodopseudomonas palustris PSB-S
Source: Sci Rep. 2018 May 9;8:7384. doi: 10.1038/s41598-018-25734-9 (PMC5943319; doi:10.1038/s41598-018-25734-9)

Cloning and characterization of a pyrethroid pesticide decomposing esterase gene, *Est3385*, from *Rhodopseudomonas palustris* PSB-S

Xiangwen Luo[1], Deyong Zhang[1], Xuguo Zhou[1,2], Jiao Du[1], Songbai Zhang[1]*, Yong Liu[1]*

[1]*Key Laboratory of Pest Management of Horticultural Crop of Hunan Province, Hunan Plant Protection Institute, Hunan Academy of Agricultural Science, Changsha 410125, China*

[2]*Department of Entomology, University of Kentucky, Lexington, KY, 40546, USA*

* E-mail: zsongb@hotmail.com (SBZ); haoasliu@163.com (YL)

Table S1 Characteristics of pyrethroids degrading proteins originated from bacteria

Protein symbol	Molecule weight (kDa)	Optimal conditions		stability		substrate sepcificity	Inhibited metal ions	*Km* (μmol·ml^{-1})	The conserved Pentapeptide motif	Esterase family	Reference
		Temperature (^{o}C)	pH	Temperature (^{o}C)	pH						
pytH	31	35	7.5	30-60	5.5-9.0	permethrin	Ag^{+},Ni^{2+}, Cu^{2+}, Hg^{2+}, Zn^{2+}	0.062±0.002	+	/	Wang et al., 2009
estI	67.5	35	7.0	30-50	6.0-8.0	permethrin	Hg^{2+},Ag^{+}	14±1.08	+	/	Choi et al., 2004
estP	73	40	7.0	40-50	5.5-9.0	permethrin	Ag^{+},Hg^{2+}	0.16±0.005	-	/	Wu et al., 2006
EstPS1	68	60	8.0	40-70	6.0-9.0	fenpropathrin		12.8	/	/	Cai et al., 2017
pytY	41.7	35	7.5	30-45	6.5-8.0	cyhalothrin	Ag^{+},Hg^{2+}	234	+	VI	Ruan et al., 2013
pytZ	25	35	7.5	30-45	6.5-8.0	cyhalothrin	Ag^{+},Hg^{2+}	265	+	VI	Zhai et al., 2012
E4	30.8	55	6.5	<55	4.5-8.5	cypermethrin	/	/	+	V	Fan et al., 2012
pye3	31.5	40	7.0	<45	5.5-9.0	Permethrin	Ag^{+},Hg^{2+}	0.18	+	I	Li et al., 2008
EstSt7	/	80	9.0	80-90	8.0-10.0	/	/	/	/	/	Wen et al., 2013
Est3385	33.94	35	6.0	15-65	5.0-8.0	fenpropathrin	Fe^{3+}, Cu^{2+}, Mg^{2+}	0.734	-	I	This study

+, existing; -, no existing; /, data not being available.

Fig. S1. Multi alignment of the sequences of pyrethroid-pesticides degrading genes. The conserved "seed" nucleotide sequences were shown as underlined ones.

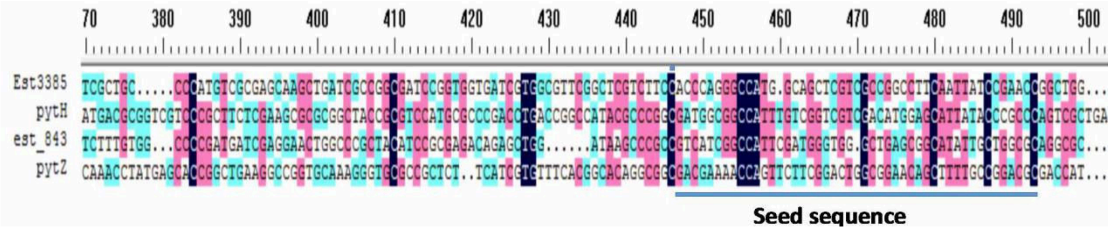

Fig. S2.Multi alignment of the amino-acid sequences of Est3385 with selected esterases. Four high identity contigs were shown with red overlines (H1~H4).

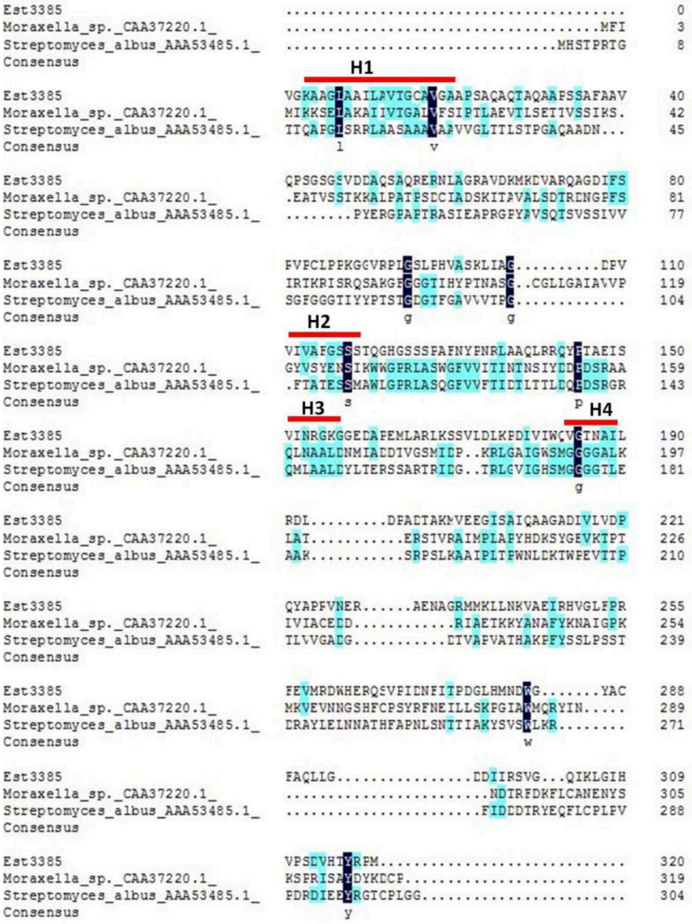

Fig. S3. **Expression, purification and western blotting of recombinant protein Est3385.** M: protein marker. Lane 1: soluble recombinant Est3385 protein in the supernatant of *E. coli* Rosetta/pGEX-3x-3385. Lane 2: purified recombinant Est3385 protein. 3: no protein control.

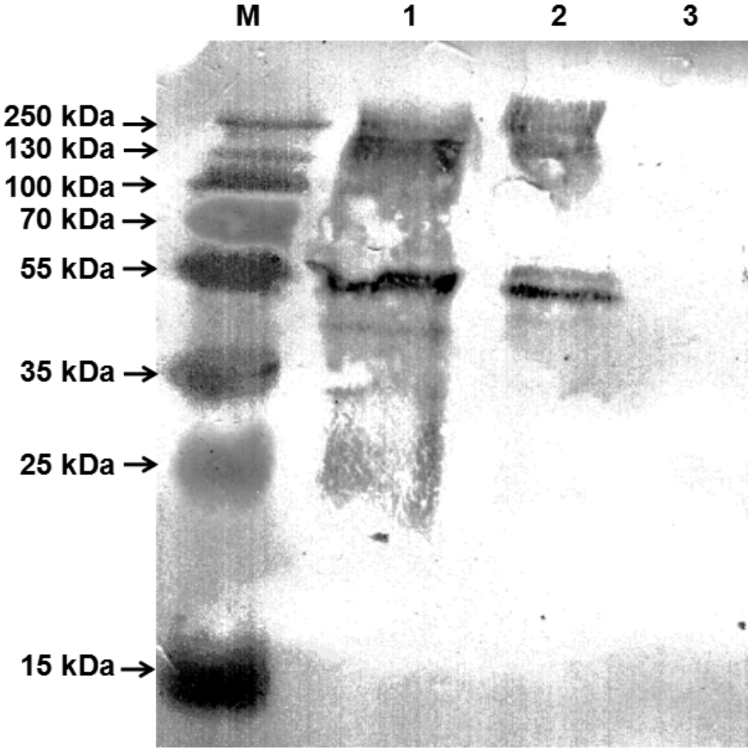

Fig. S4. Quantification of fenpropathrin residue by gas-chromatography (GC). (Reaction mixture including 100 μg·ml^{-1} of fenpropathrin within PBS buffer. **Est3385**: reaction mixture adding 1 μg·ml^{-1} Est3385; **CK1 (GST)**: reaction mixture adding 1 μg·ml^{-1} glutathione S-transferase; **CK2 (No protein)**: reaction mixture adding equivalent volume of PBS buffer.)

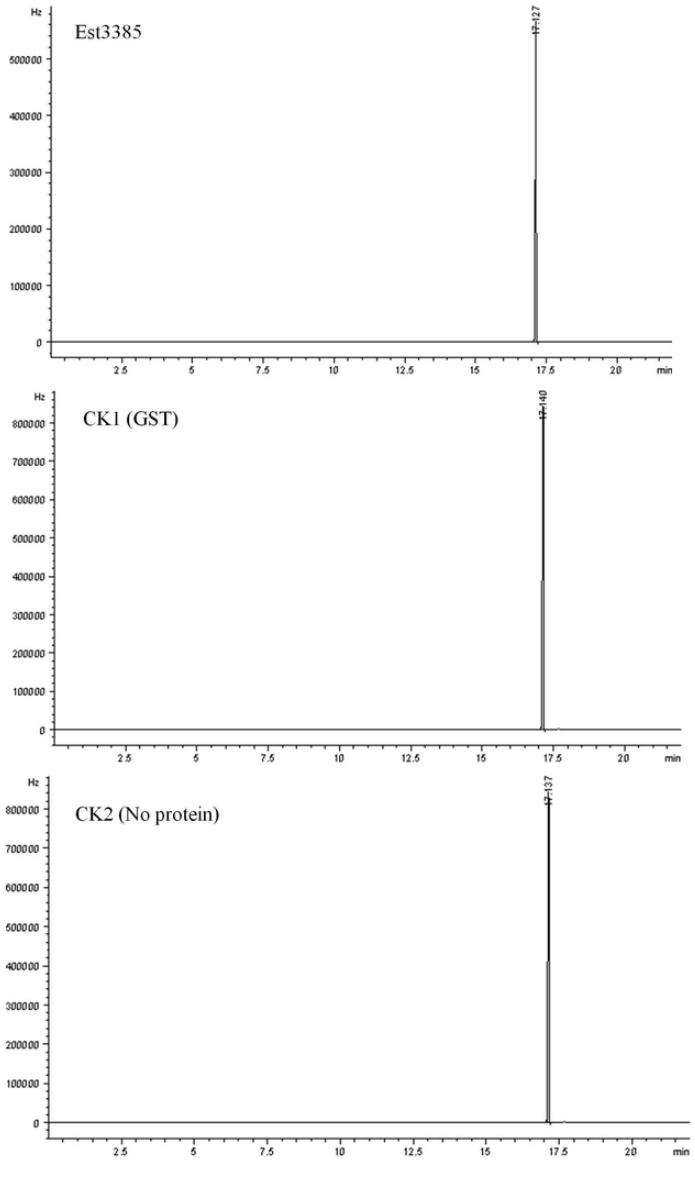

Fig. S5. Degradation of ρ-nitrophenyl esters and short-chain fatty acids by Est3385. The substrate activity was set as 100% when ρ-nitrophenyl acetate and Formic acid was used as the substrate. (Reaction mixture including 20 μg·ml^{-1} ρ-nitrophenyl esters and fatty acids, 1 μg·ml^{-1} Est3385. A: Degrading efficiency of ρ-nitrophenyl esters by Est3385; B: Degrading efficiency of short-chain fatty acids.)

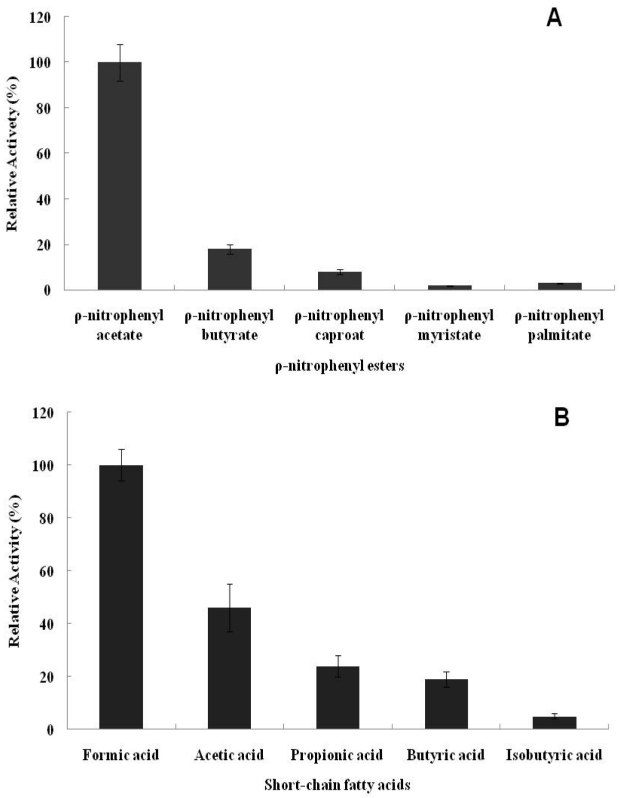

Fig. S6. The protein structure of Est3385. A: 3D structure of protein Est3385; B: Amino acid residues Ser117-119; C: Amino acid residues Ser125-128.

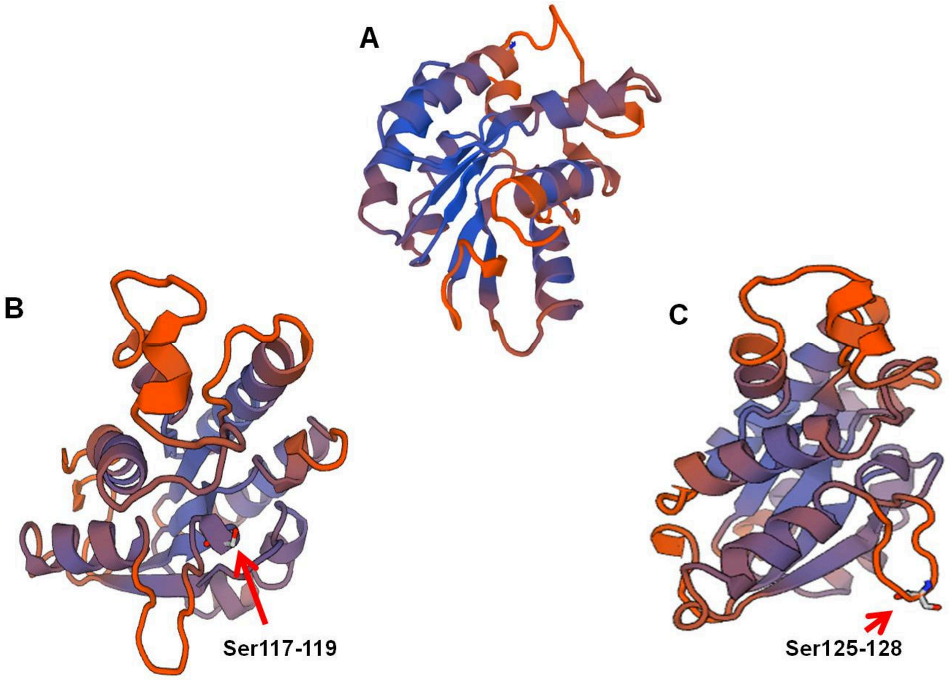

Fig. S7. The kinetic constant of protein Est3385 degrading fenpropathirn (Michaelis-Menten equation). [V]: degrading velocity (U·μg^{-1}); [S]: fenpropathrin initial concentration (μg·μl^{-1}).

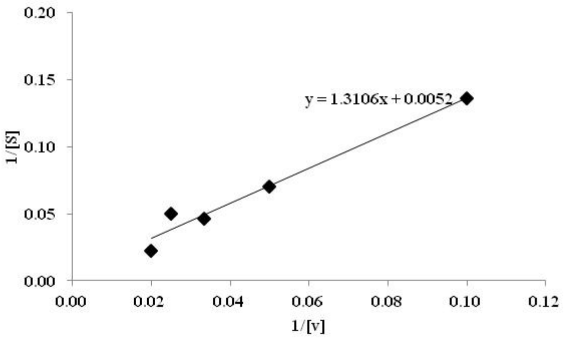

Supplement: Supplementary file 1 — dataset 1 [file 41598_2018_25734_MOESM1_ESM.pdf]
